# Instability of the posterior pelvic ring: introduction of innovative implants

**DOI:** 10.1186/s13018-021-02770-2

**Published:** 2021-10-18

**Authors:** Niklas Grüneweller, Dirk Wähnert, Thomas Vordemvenne

**Affiliations:** Department of Trauma Surgery and Orthopedics, Protestant Hospital of Bethel Foundation, University Hospital OWL of Bielefeld University, Campus Bielefeld-Bethel, Burgsteig 13, 33617 Bielefeld, Germany

**Keywords:** Dorsal pelvic ring, Instability, Iliosacral joint, Osteoporotic fracture

## Abstract

**Background:**

Increasing numbers of posterior pelvic ring fractures, especially in elderly patients, demonstrate the need for soft tissue protecting surgical techniques. Standard of care is iliosacral screw osteosynthesis. This type of osteosynthesis has its limitations especially in patients with reduced bone properties. Therefore, the development of new and straightforward surgical techniques and implant designs is favorable.

**Methods:**

Introducing this modular system for the posterior pelvic ring, known complications of iliosacral screw osteosynthesis, such as implant loosening and malpositioning may be reduced, due to innovative mechanical characteristics.

**Results:**

The shown cases demonstrate the potential benefits of the system with a wide range of treatment options due to its modularity.

**Conclusion:**

The modular implant system presented here can significantly facilitate and improve the stabilization of posterior pelvic ring instabilities.

## Background

Fractures of the pelvis are rare overall, accounting for 2–8% of all fractures. However, several studies show that their proportion is increasing significantly in elderly patients [1–4]. In this patient population, fragility fractures of the pelvis occur after minor trauma or even without a trauma event [5]. The incidence of fragility fractures of the posterior pelvic ring increases from 92 to 446 per 100.000 in the population aged over 65 and over 85, respectively [6]. Analyses of the German pelvic registry showed that the fracture type is underestimated, especially in these patients, so that recommendations for structured diagnostics of geriatric pelvic fractures were developed [7–10]. The operative stabilization of fragility fractures of the posterior pelvic ring is recommended in patients suffering from immobilizing or prolonged pain or progressive fracture displacement [5, 11]. For the treatment of fragility fractures of the posterior pelvic ring, iliosacral screw osteosynthesis can be considered the gold standard because it is a minimally invasive, soft-tissue-preserving, and overall safe procedure. However, the reduced bone quality in the sacrum in patients with fragility fracture significantly reduces implant anchorage and increases the risk of implant failure [12, 13]. Screw loosening, turn-out, and cutting-out of the screws are the main events observed [14–16]. In the literature, the rate of screw loosening is reported to be as high as 20% in patients with fragility fractures of the pelvis [17]. In recent years, new methods and implants have been introduced to specifically address these problems in the surgical stabilization of posterior pelvic ring fractures [18–26]. However, significant aspects remain controversial, for example, cement augmentation can improve screw anchorage, but cannot prevent turn-out [19]. Procedures, such as transiliac bridging create greater soft tissue damage and may cause discomfort for the patient [18, 24, 25]. The transsacral bar is only applicable when the morphology of the sacrum is appropriate [26].

To the best knowledge of the authors, no implant system exists to address all entities of dorsal pelvic ring instabilities with one modular system. Therefore, this paper wants to present a new modular implant system and its concept for the treatment of fractures of the posterior pelvic ring based on the first patient cases.

## Methods

The new Verticale® implant system consists of two modules: (1) a iliosacral screw with washer/ washer plate and (2) a modular triangular fixation system. The implants are manufactured by Silony (Silony Medical GmbH, Leinfelden-Echterdingen, Germany). All implants are made of titanium alloy (Ti6Al4V ELI) and are certified and approved for clinical use.

### Iliosacral screw with washer and washer plate

**Fig. 1:**
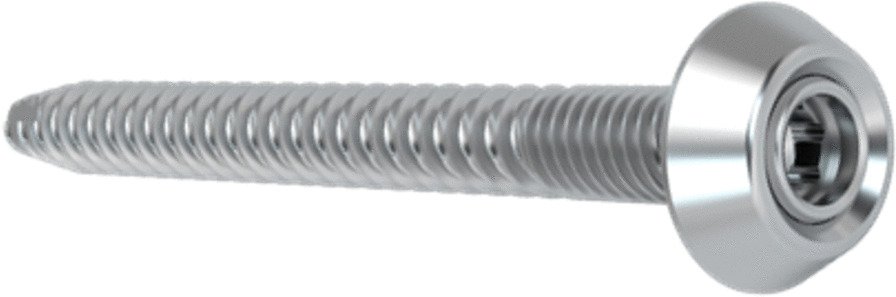
Picture of the iliosacral screw with a diameter of 7.2 mm, a two-piece thread and a pre-mounted and fixed, but movable washer

The iliosacral screw has a diameter of 7.2 mm and is cannulated (Fig. 1). It is characterized by a two-piece thread with a large thread pitch over the anterior 3/4 of the screw length and a small thread pitch in the area of the 1/4 screw head proximity. Furthermore, the screw has a pre-mounted and fixed, but movable washer with 17 mm in diameter. This design allows optimal compression of the fracture zone and safe handling during screw insertion and removal. With its larger washer surface, the risk of ilium penetration is reduced. In particular, this screw is an option in the treatment of the posterior pelvic ring in patients with good bone quality.

**Fig. 2:**
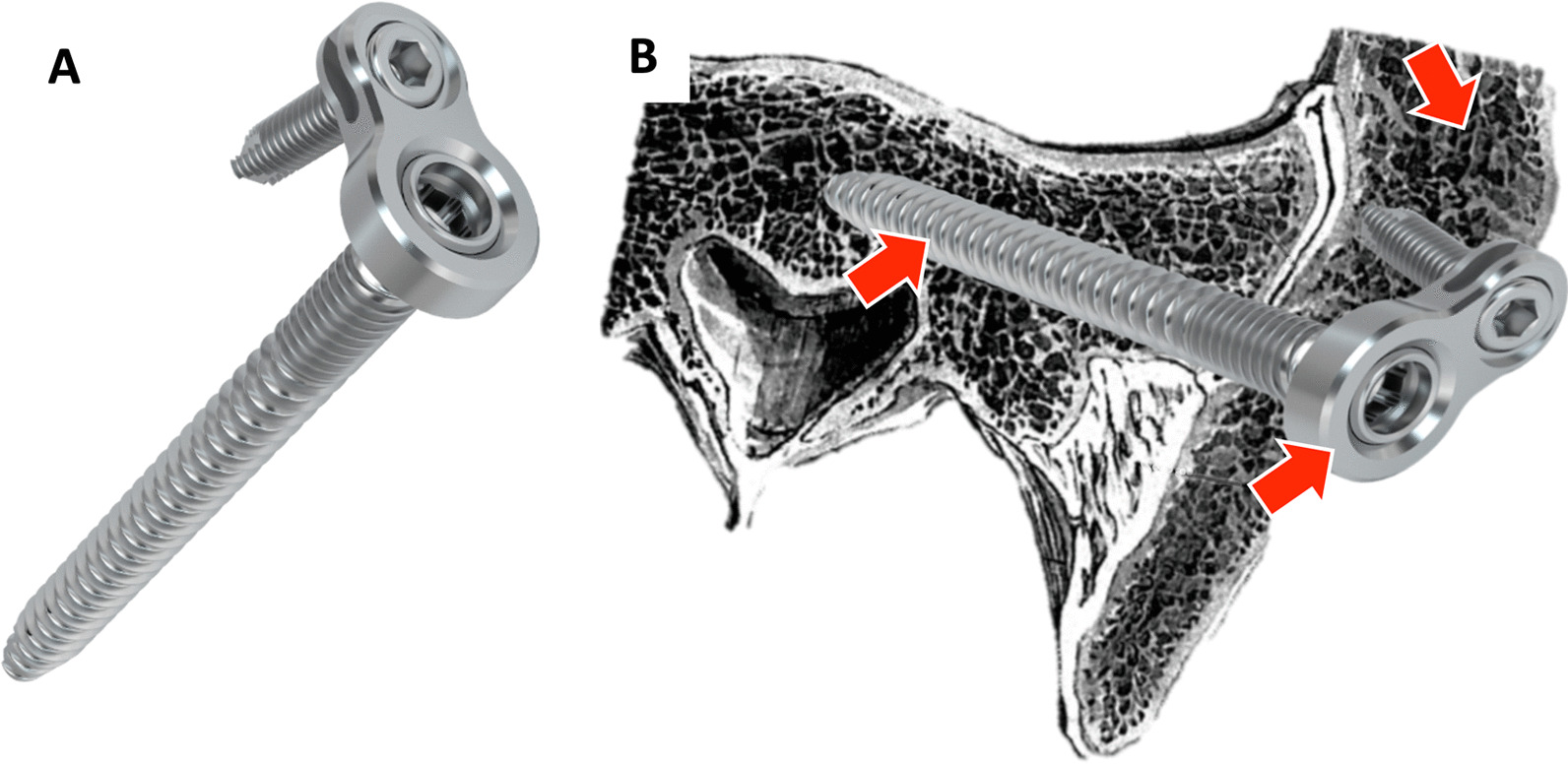
**a** Iliosacral screw with attached washer plate and ilium locking screw. **b** Shows the 3-point support mechanism with red arrows

The combination of the iliosacral screw with a pre-mounted and fixed, but movable washer plate allows the additional placement of an angular stable screw in the ilium 360° around the head of the iliosacral screw (Fig. 2a). This combination is intended to increase primary construct stability and to provide effective protection against screw loosening and turn-out. Additionally, the larger washer plate surface also reduces the risk for washer penetration. Furthermore, the 3-point support of the construct (Fig. 2b) prevents the mechanism of screw cut-out. In addition, this screw offers the possibility of additive cement augmentation and can therefore effectively be used in cases of osteoporotic posterior pelvic ring fractures.

### Verticale®—triangular fixation system

**Fig. 3:**
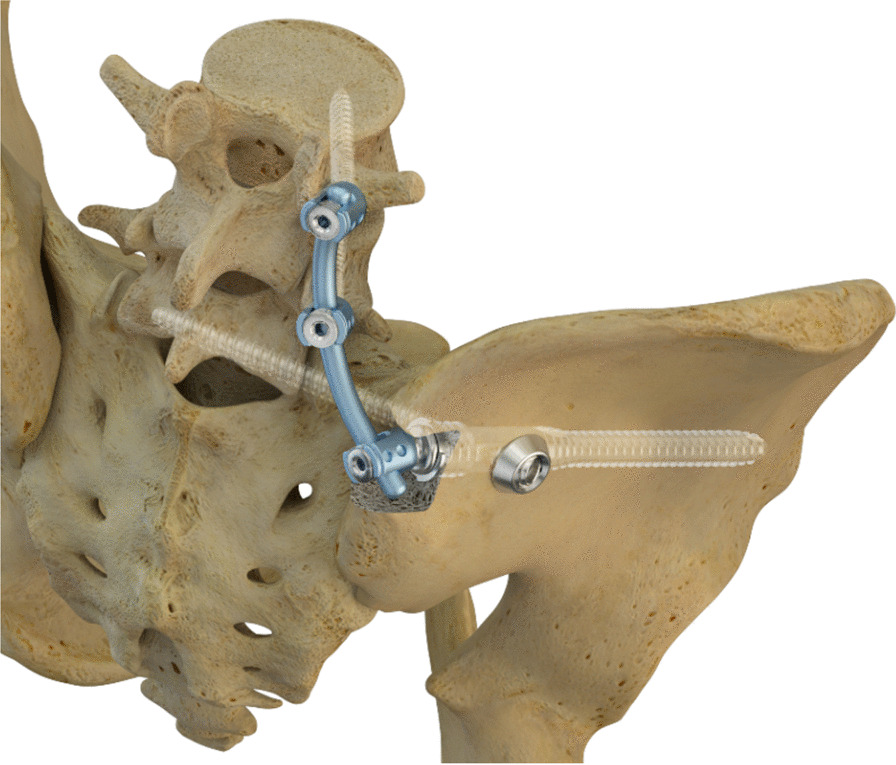
Picture showing the Verticale®—Triangular Fixation System

The triangular fixation system is a system of modular design, which allows triangular ilium-iliosacral stabilization as well as extension to lumbo-pelvic stabilization (Fig. 3).

**Fig. 4:**
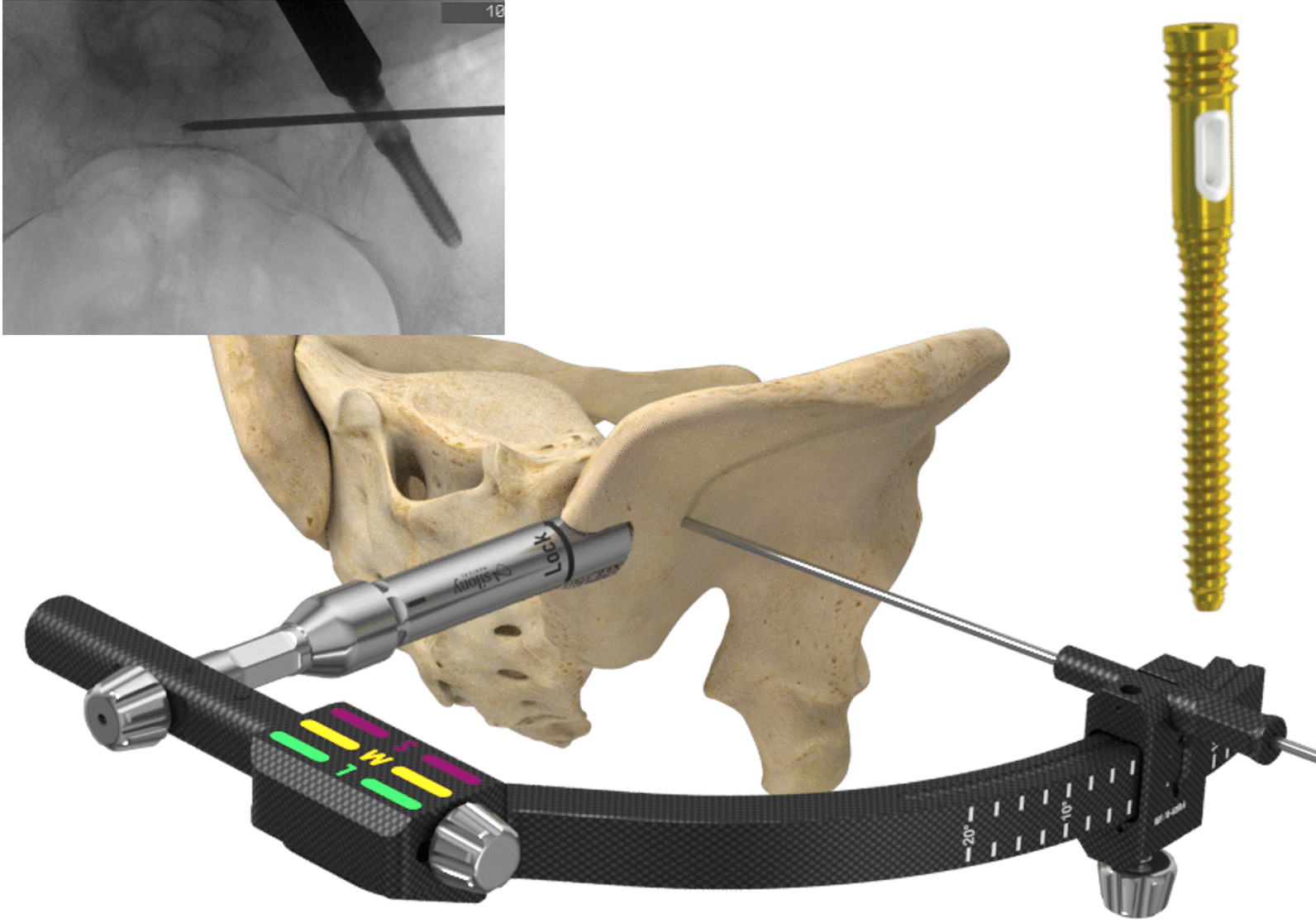
Picture showing the ilium screw design and the insertion of the ilium screw and the attached guiding device for the placement of the iliosacral screw with the guide wire already in place (x-ray)

**Fig. 5:**
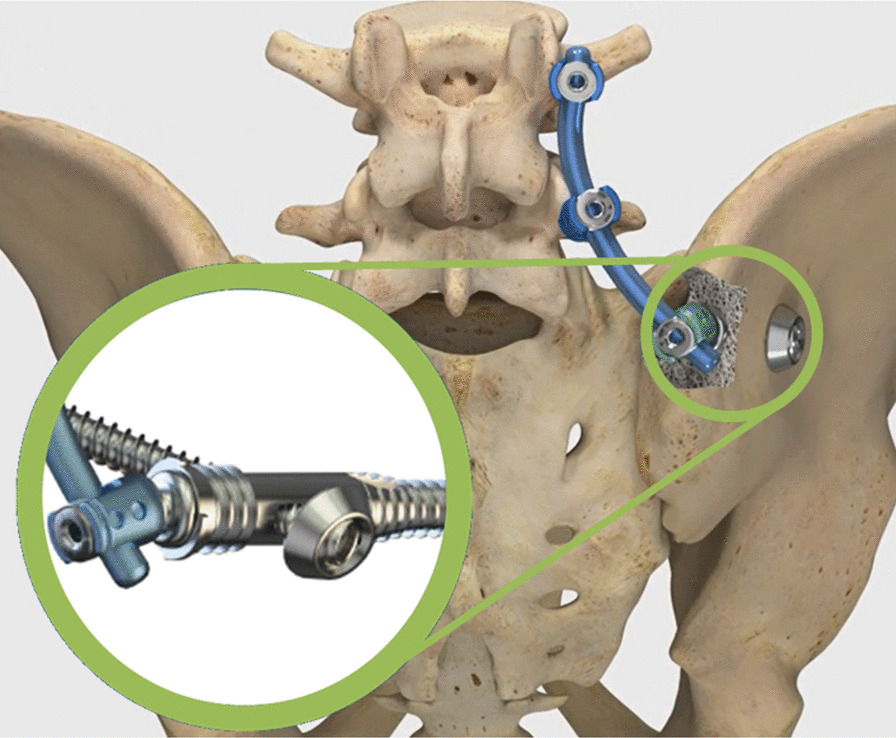
Triangular fixation including the extension to lumbo-pelvic stabilization

In this system, triangular ilium-iliosacral stabilization is achieved by means of a special ilium screw with longitudinal hole for the iliosacral screw (Fig. 4). The ilium screw is inserted minimally invasively, and the iliosacral screw is placed over an adjustable in-sertion guide (Fig. 4). The longitudinal hole allows angular stable placement of the iliosa-cral screw. The design of the head of the ilium screw allows easy and minimally invasive extension to lumbo-pelvic stabilization (Fig. 5). Furthermore, with bilateral instrumentation, the connection of both ilium screws is possible in the sense of transiliac bridging. This system is indicated for instabilities of the dorsal pelvic ring, including the iliosacral and the lumbar-pelvic region with all grades of instability (due to trauma, tumor, osteoporosis).

## Results

### Iliosacral screw with washer and washer plate

The procedure of stabilizing a fracture of the posterior pelvic ring using the iliosacral screw with washer plate follows the surgical standards of iliosacral screwing. The patient can be placed in supine as well as prone position. The coverage of the surgical area is based on the standard, e.g., for an intraoperative 3D scan or navigation. The implantation follows a wire-guided technique, like in standard iliosacral screws. With approximately 3 cm, the incision is slightly larger compared to a standard iliosacral screw. This is necessary to insert the washer plate with its two trocars (one for the screwdriver of the iliosacral screw and one for the implantation of the ilium locking screw). The design of the screw with its two-piece thread results in compression between the sacrum and the iliac portion of the fracture, recognizable by the high torque required to screw in the last 2 cm. Care must be taken to define and check the position of the washer plate and thus the ilium locking screw before tightening the iliosacral screw to achieve sufficient washer plate orientation.

**Fig. 6:**
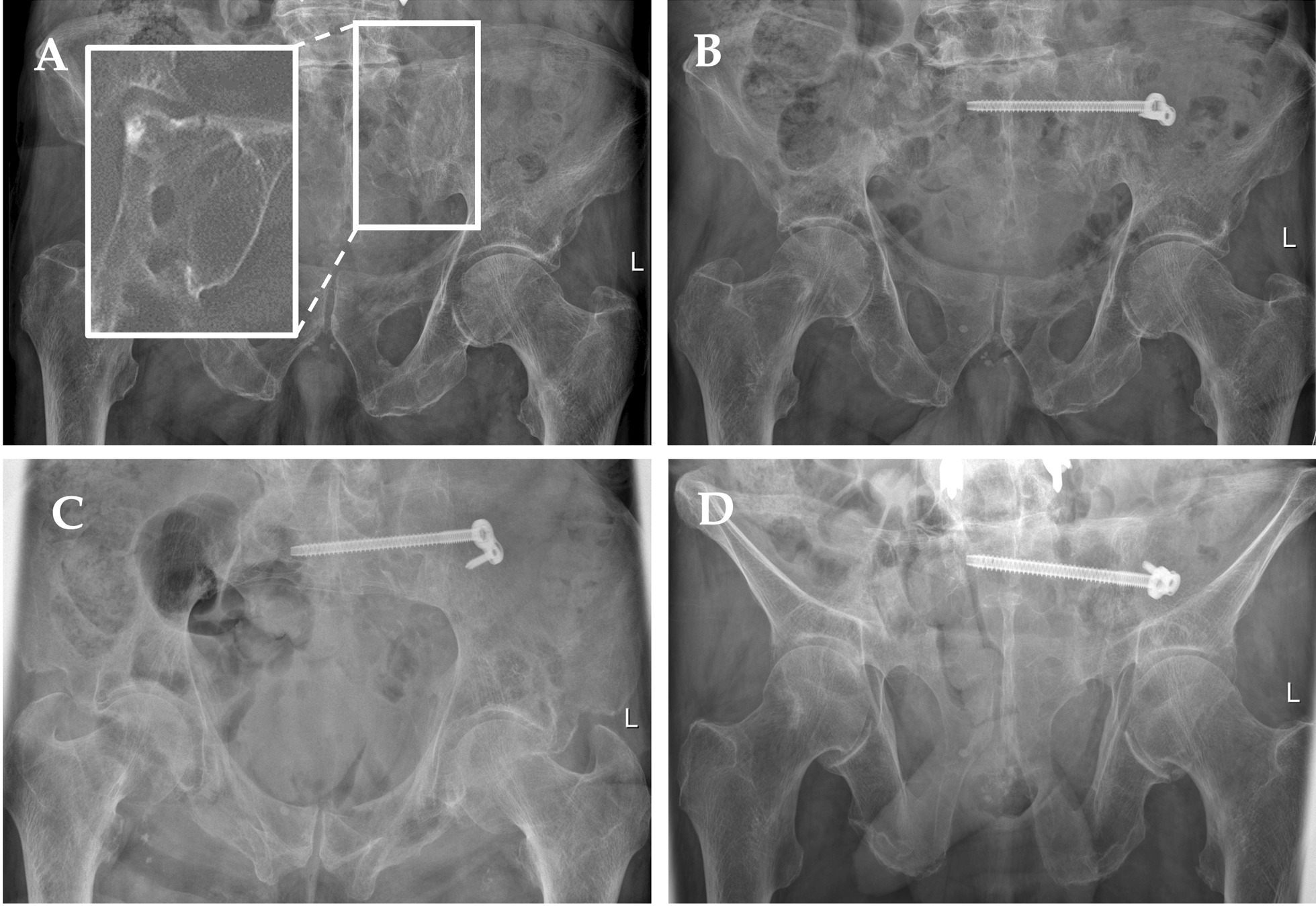
Case of a FFP type IIb fracture in an 81-year-old male patient after a fall from a standing position. **a** Initial x-ray of the pelvis in anterior–posterior view with a magnification of the left sacrum from the CT-scan. **b** Post-operative x-ray control in anterior–posterior direction after fracture stabilization using the iliosacral screw with washer plate and angular stable ilium screw. **c** Inlet-view. **d** Outlet-view

**Fig. 7:**
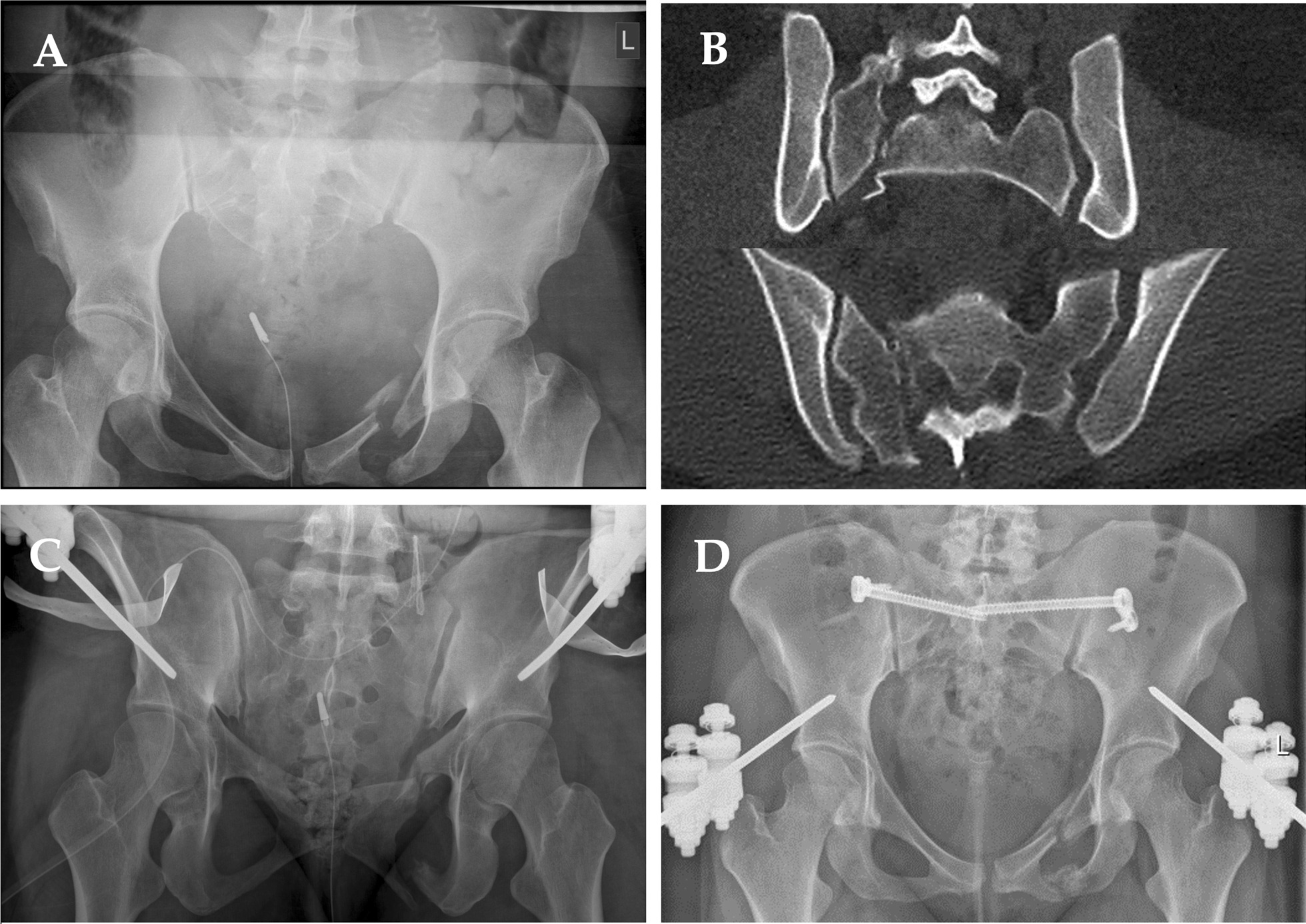
Case of a AO Typ C pelvic fracture (AO 61 C3.2) in a 30-year-old female after car accident. **a** Initial x-ray of the pelvis in the emergency department showing the injury of the anterior and posterior pelvic ring. **b** Two slices from the CT-scan showing the fracture of the sacrum on the one and the rupture of the iliosacral joint on the other side. **c** X-ray control after initial stabilization with a supraacetabular fixateur. **d** X-ray of the pelvis after stabilization of the dorsal pelciv ring using bilateral iliosacral screws with washer plates

Figures 6 and 7 demonstrate the iliosacral screw with washer plate using two case studies.

### Triangular fixation

**Fig. 8:**
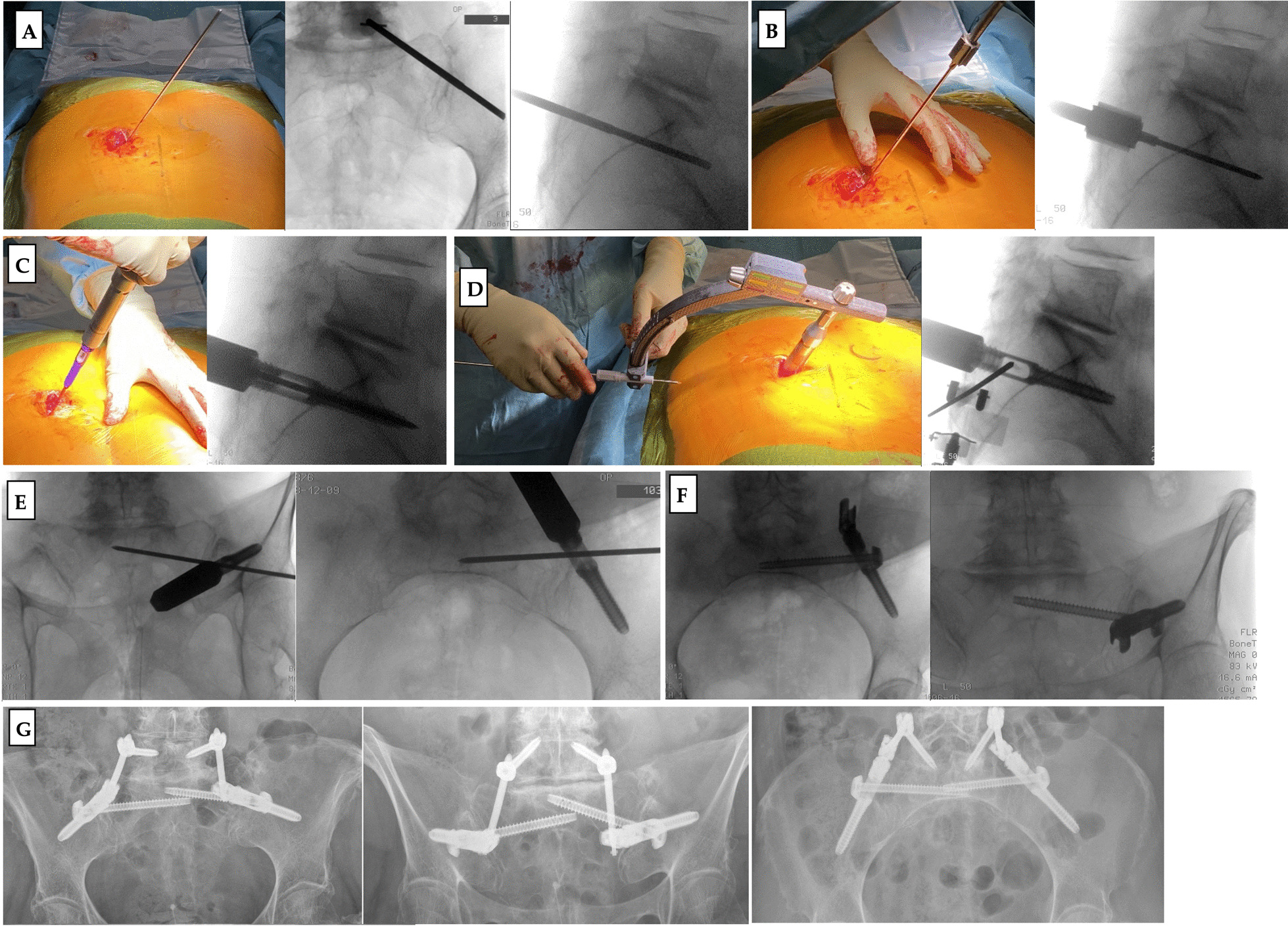
Pictures showing the operative case of 72-year-old female patient with a U-shaped sacrum fracture after a fall from standing hight. **a** Patient in prone position with the Kirschner wire for the ilium screw in position, x-rays showing correct placement. **b** Preparation of the entry point of the ilium screw with size determination in lateral x-ray projection. **c** Insertion of the ilium screw and control of the correct insertion depth with the image intensifier. **d** Attachment of the guiding device and placement of the Kirschner wire for the iliosacral screw under x-ray control. **e** Checking final Kirschner wire position in two planes using the image intensifier. **f** X-ray control after iliosacral screw insertion and adaption of the polyaxial head onto the ilium screw for lumbo-pelvic stabilization. **g** Postoperative x-ray control after bilateral triangular fixation and lumbo-pelvic stabilization

The triangular fixation using the presented implants also follows surgical standard procedures at the dorsal pelvic ring. The patient is placed in prone position, and important anatomical landmarks are marked (e.g., lumbar spinous processes and the spina iliaca posterior superior, Fig. 8a). Furthermore, it is crucial to check that the patient positioning allows undisturbed intraoperative imaging (if necessary also 3D scan, navigation).

First, the ilium screw is placed over the posterior iliac crest. Therefore, a guide wire is inserted in the correct position (Fig. 8a). The entry point for the ilium screw is then prepared with an awl, determining the correct size of the ilium screw (Fig. 8b). This is done by choosing the size of the awl, the tip must reach the contour of the posterior edge of the first sacral vertebra in the lateral x-ray. The ilium screw is then inserted, again care must be taken to ensure the correct insertion depth so that the longitudinal hole aligns with the posterior edge of the first sacral vertebra, and sufficient dorsal soft tissue coverage can be achieved (Fig. 8).

The insertion handle is then connected for placement of the iliosacral screw. With the help of this insertion handle, a guide wire for the iliosacral screw is placed, and the insertion handle is corrected until the wire has reached the desired position. The cortex of the ilium is then opened with an awl. A drill must not be used, as this would destroy the PE inlet of the ilium screw. Due to the PE (polyethylene) insert in the longitudinal hole of the ilium screw, an angular stable connection between ilium and iliosacral screw is achieved. After removal of the insertion handle, either a closing cap or a polyaxial screw head for extension to the lumbar spine is placed onto the ilium screw. This allows for minimally invasive spinopelvic stabilization.

The insertion handle works monoaxially in direction of the ilium screw trajectory. This simplifies the iliosacral wire insertion compared to free hand technique with higher uncontrollable variability. For change in inclination, the ilium screw has to slightly turned in or out, which changes the orientation of the longitudinal hole. Thus, the risk of implant malpositioning can be reduced.

The unique ilium screw design with its longitudinal hole solves the problem of corridor overlapping of the ilium screw and the sacroiliac screw in standard triangular fixation.

## Discussion

This work presents new implants for the minimally invasive stabilization of the posterior pelvis ring. The modular design of the implant system allows gradual expansion from the simple iliosacral screw to spino-pelvic stabilization. The system presented here addresses several weaknesses in the surgical management of posterior pelvic ring injuries.

The system was successfully used in the first clinical applications. All components of the modular system were implanted. The indication and preoperative planning appear to be important. For the iliosacral screw fixation and the triangular fixation, the bony corridors must be checked in the cross-sectional imaging. For triangular screw fixation, the iliosacral screw is placed using an insertion guide. This procedure has proven to be very practicable in clinical use, as the guide wire is guided in a sleeve and can be precisely corrected level by level via the insertion guide. Furthermore, all instruments are optionally prepared for the use of 3D image-guided navigation in addition to the conventional X-ray guided procedure, which can be recommended for difficult anatomical conditions in the S1 sacral region. Another relevant topic in this anatomical region is soft tissue problems. The ilio-sacral screw is not critical, as there is always sufficient soft tissue coverage. The head of the ilium screw is placed below the level of the bone, for which the posterior iliac crest is prepared with an awl. The different length of the iliac screw in the head region ensures both an optimal fit on the iliac crest and correct positioning of the crossing oval hole for the iliac screw. In addition, the correct fit of the iliac screw is important for the biomechanical anchorage of the construct. In this way, adequate soft tissue coverage can be ensured during triangulation stabilization. The lumbo-pelvic fixation is performed minimally invasive to ensure wound healing. Nevertheless, regular critical wound checks, and dressing changes are necessary after these operations.

In a recently published paper, Zderic et al. present a new implant prototype for stabilization of dorsal pelvic ring injuries [22]. In a biomechanical study, they compared the standard iliosacral screw and the transsacral stabilization using an artificial bone model with a sacral osteotomy. The new concept represents a screw-in-screw fixation and is thus similar to the washer plate presented in this article. The authors were able to show significantly less interfragmentary movement and significantly less implant motion for the prototype compared to the standard screw. Furthermore, the screw-in-screw stabilization was able to completely prevent the iliosacral screw from turning out [22].

The potential of triangular fixation was investigated by Schildhauer et al. in a bio-mechanical work on human pelvises with transforaminal sacral fracture [27]. The authors were able to show a significantly higher initial stability of triangular fixation compared to iliosacral screw fixation. Furthermore, interfragmentary deformation at maximum load remained constant with triangular stabilization after 10,000 cycles, whereas it doubled with iliosacral screw fixation in relation to the first load cycle and quadrupled in relation to triangular stabilization [27].

## Conclusion

The modular implant system presented here can significantly facilitate and improve the stabilization of posterior pelvic ring instabilities. Nevertheless, further biomechanical and clinical investigations will highlight the potential of the presented implant system.

## Data Availability

Data sharing is not applicable to this article as no datasets were generated or analyzed during the current study.
